# Barriers and Facilitators to Implementation of Antibiotic Stewardship Programmes in Hospitals in Developed Countries: Insights From Transnational Studies

**DOI:** 10.3389/fsoc.2020.00041

**Published:** 2020-07-08

**Authors:** Magdalena Rzewuska, Eilidh M. Duncan, Jill J. Francis, Andrew M. Morris, Kathryn N. Suh, Peter G. Davey, Jeremy M. Grimshaw, Craig R. Ramsay

**Affiliations:** ^1^Health Services Research Unit, University of Aberdeen, Aberdeen, United Kingdom; ^2^School of Health Sciences, City University of London, London, United Kingdom; ^3^Department of Medicine, Sinai Health System and University Health Network, Toronto, ON, Canada; ^4^Department of Medicine, Faculty of Medicine, University of Toronto, Toronto, ON, Canada; ^5^Department of Medicine, The Ottawa Hospital and University of Ottawa, Ottawa, ON, Canada; ^6^Ottawa Hospital Research Institute, Ottawa, ON, Canada; ^7^Division of Population Health & Genomics, Medical School, University of Dundee, Dundee, United Kingdom; ^8^Clinical Epidemiology Program, Ottawa Hospital Research Institute, Ottawa, ON, Canada; ^9^Department of Medicine, University of Ottawa, Ottawa, ON, Canada

**Keywords:** antimicrobial stewardship, systematic review, barriers and facilitators, theoretical domains framework, hospitals, behavior change

## Abstract

**Objectives:** To identify perceived influences on implementation of antibiotic stewardship programmes (ASPs) in hospitals, across healthcare systems, and to exemplify the use of a behavioral framework to conceptualize those influences.

**Methods:** EMBASE and MEDLINE databases were searched from 01/2001 to 07/2017 and reference lists were screened for transnational studies that reported barriers and/or facilitators to implementing actual or hypothetical ASPs or ASP-supporting strategies. Extracted data were synthesized using content analysis with the Theoretical Domains Framework as an organizing framework. Commonly reported influences were quantified.

**Results:** From 3,196 abstracts 75 full-text articles were screened for inclusion. Eight studies met the eligibility criteria. The number of countries involved in each study ranged from 2 to 36. These studies included a total of 1849 participants. North America, Europe and Australasia had the strongest representation. Participants were members of special interest groups, designated hospital representatives or clinical experts. Ten of the 14 theoretical domains in the framework were present in the results reported in the included studies. The most commonly reported (≥4 out of 8 studies) influences on ASP implementation were coded in the domain “environmental context and resources” (e.g., problems with data and information systems; lack of key personnel; inadequate financial resources) and “goals” (other higher priorities).

**Conclusions:** Despite an extensive transnational research effort, there is evidence from international studies of substantial barriers to implementing ASPs in hospitals, even in developed countries. Large-scale efforts to implement hospital antibiotic stewardship in those countries will need to overcome issues around inadequacy of information systems, unavailability of key personnel and funding, and the competition from other priority initiatives. We have enhanced the evidence base to inform guidance by taking a behavioral approach to identify influences on ASP uptake.

**Systematic review registration**: PROSPERO registration number CRD42017076425.

## Introduction

Overuse or inappropriate use of antibiotics is a key driver of the worldwide escalation of antibiotic resistance (Carlet et al., 2011), which is a major threat to global public health and patient safety. Antibiotic resistance is associated with excess mortality and morbidity, prolonged hospital stay and increased health care costs (De Kraker et al., 2011). Antibiotic resistance has predominantly been a clinical problem in hospital settings (Llor and Bjerrum, 2014), which are particularly susceptible to harboring multidrug-resistant organisms (Chemaly et al., 2014).

Transnational response to this global crisis have been co-ordinated by a World Health Organization Global Action Plan (World Health Organization, 2015) and through a strategic research agenda on antibiotic resistance, which currently unites 28 partners globally (JPIAMR, 2017). The global action plan sets out five strategic objectives (World Health Organization, 2015): (1) improve awareness and understanding of antibiotic resistance; (2) strengthen knowledge through surveillance and research; (3) reduce the incidence of infection; (4) optimize the use of antibiotic agents; and (5) ensure sustainable investment in countering antibiotic resistance. A key approach to optimizing the use of antibiotics is the deployment of antibiotic stewardship programmes (ASP) in hospitals. An ASP involves a team that implements a coherent set of actions that promotes the responsible use of antibiotic agents (Dyar et al., 2017).

Effectiveness of ASPs implemented by hospitals is likely to differ depending on both ASP elements and contextual factors. In practice, ASP initiatives are a heterogeneous group of system- and organization-based strategies and actions (Dyar et al., 2017), and countries and organizations may vary greatly in their capacity to deploy the necessary resources to implement those interventions (Tiong et al., 2016). For example, there is substantial transnational and even national variability in appropriate prescribing and compliance with antibiotic guidelines (Sandora et al., 2016; Turnidge et al., 2016; Dentan et al., 2017; Mousavi et al., 2017). The international research community faces the challenge of optimizing implementation initiatives, such as ASPs, by producing generalisable evidence that incorporates relevant theory and an understanding of the contextual influences (Ivers and Grimshaw, 2016).

Amongst the key research gaps identified in the WHO action plan is the need to understand the behaviors required to support effective ASPs (World Health Organization, 2015). The difference between recommendations for appropriate antibiotic use (the “what”) and behavioral change interventions (the “how”) is key (Hulscher and Prins, 2017). ASPs require clinicians to change their behaviors. There is a wealth of theoretical and empirical evidence from the behavioral sciences about how to change behavior, yet this is currently underutilized in antibiotic stewardship studies (Charani et al., 2011; Rawson et al., 2017). Hence there are opportunities to enhance the effects of ASPs using behavioral approaches (Davey et al., 2017). Methods and tools from the behavioral sciences should be used to select the most promising interventions to change behavior, based on a careful assessment of barriers and facilitators to practice change (Davey et al., 2017; Hulscher and Prins, 2017). To date, one systematic review has explored the evidence on barriers and facilitators of antibiotic prescribing behavior in acute care (Charani et al., 2011); however, an evidence synthesis using behavior change theory to identify influences on implementation of ASPs is lacking.

### Aims and Objectives

The aim of this study was to inform the development of large-scale contextually optimized quality improvement hospital ASPs, by improving the understanding of contextual influences on ASP implementation, through the framework of identifying “barriers and facilitators.” The objectives were:

1. To conduct a systematic review of transnational research to identify commonly perceived barriers and facilitators to implementation of actual or hypothetical ASPs in hospitals.
2. To provide an exemplar of the use of a behavioral framework analysis to conceptualize identified barriers and facilitators to ASPs in hospitals.

## Methods

This systematic review was conducted in accordance with the Center for Reviews and Dissemination's guidance for undertaking reviews in healthcare (Centre for Reviews Dissemination, 2009) and reported adhering to the PRISMA guidelines (Liberati et al., 2009).

### Search Strategy

A search strategy was developed by an Information Specialist in collaboration with the review authors, who generated a list of possible relevant keywords related to antibiotic stewardship, hospital settings and national or international study scope. The search strategy was not intended to be restrictive to a specific study design, but excluded studies on animals, and editorials and abstracts. The review team screened a random sample of 100 identified abstracts to verify if relevant studies were identified. Based on the results of this verification and a study protocol, the search string was amended so that the search became more sensitive to data on barriers and facilitators and infection control and antibiotic policies research. A new search was performed and achieved satisfactory comprehensiveness such that no further amendments were applied. The final search strategy can be found in the protocol.

### Data Sources

An initial scoping search for published literature was performed using the Medline and EMBASE electronic bibliographic databases. There was no start date limit; the EMBASE and Medline databases were searched from 1980 and 1946 respectively to 18th July 2017. We observed a sudden increase in numbers of identified studies published after 2000. The first global strategy to lead the response of 193 United Nation states to antibiotic resistance was developed by the WHO in 2001 (World Health Organization, 2001). The first guideline for designing an ASP was published by the Society for Healthcare Epidemiology of America (SHEA) and Infectious Diseases Society of America (ISDA) in 2007 (Dellit et al., 2007). Consequently, we included only the papers published between 2001 and 18th July 2017. References of retrieved articles, systematic reviews and personal files were searched for relevant studies.

### Study Selection

A random sample of 100 titles and abstracts was double screened by two reviewers (EMD and MR) to assess consistency, and revisions were made to the definitions and criteria to enhance clarity. In the next, single screening phase, the same two reviewers independently screened the remaining titles and abstracts using an Excel spreadsheet. For record keeping purposes, we documented details of excluded abstracts, including topic, scope (country-level or international), setting, participants and design. Five randomly selected full-text articles were double screened for inclusion by two reviewers (MR, CRR) to assess reliability. Disagreements were resolved by discussion and the remaining selected full-text articles were assessed by one reviewer (MR), with any uncertainties related to eligibility of a specific article resolved by discussion with a second reviewer (CRR or EMD).

### Inclusion Criteria

Studies were included in the systematic review if they met the following eligibility criteria:

- Transnational studies, i.e., in which participants were from more than one country, were included.
- Studies on ASPs or specific antibiotic stewardship strategies used to support ASPs, such as selective reporting of antibiotic susceptibility test (AST) results (a laboratory-based ASP intervention which consists of reporting to prescribers only few antibiotics or not reporting at all when colonization is likely) (Barlam et al., 2016).
- Reported primary data published in full-text articles, from structured (e.g., questionnaires with specific response formats) and semi-structured (open-ended questions) methods of inquiry.
- Settings included hospital inpatient care settings or mixed hospital inpatient and outpatient settings.
- Reported barriers and/or facilitators to implementing an ASP. Studies which did not use the terms “barriers” and “facilitators” explicitly were included when they used associated terms such as “issues,” “difficulties,” “problems with,” “(in)adequacy of support for an ASP,” “obstacles,” “enablers,” “solutions.”
- There were no restrictions for languages.

### Exclusion Criteria

We excluded review articles, guidelines, studies focused on antimicrobials other than antibacterials (i.e., antituberculous, antifungal, antiparasitic, antiviral drugs), and studies of patients from ambulatory care or long-term healthcare settings.

### Data Extraction

For each included study, one reviewer (MR) completed data extraction using a data extraction spreadsheet to include the following information: methods (author, study design, study response rate), population (country, type of hospital setting and participants, sample size), description of an ASP; barriers and facilitators to implementation (a method of assessment, response rate to a question, results verbatim including type and quantification (e.g., rates or ranks). A second reviewer (EMD) double-checked the extracted information. In one case a study author was contacted via e-mail to obtain additional information that was not reported in the published article.

### Appraisal of Methodological Quality of Included Studies

We used relevant parts of the Mixed Methods Appraisal Tool (MMAT) (Pluye et al., 2011) to conduct quality appraisal. Specifically, if the research question was judged to be clear and the data collection methods were judged to be appropriate, further methodological appraisal was undertaken. For quantitative studies, four criteria (sampling strategy, representativeness, appropriateness of measurement and response rate) were applied. Two reviewers (EMD and MR) independently assessed the methodological quality of each included study by scoring each study against each MMAT item with the following nominal scale: yes (clearly met), no (clearly not met) and unclear (not clear if met) resolving disagreements by discussion.

### Data Synthesis and Presentation

Qualitative analysis was conducted using theory-based content analysis, which involves a directed approach to content analysis (a systematic method of making specific inferences from differential levels of text) (Hsieh and Shannon, 2005). Levels of text can be broadly divided into primary content (i.e., themes and main ideas of the text) and latent content (i.e., context information) (Vaismoradi et al., 2013). We applied the Theoretical Domains Framework (TDF), a theoretical framework, developed by synthesizing behavioral theories through a systematic consensus process, as a framework for investigating the barriers and facilitators to behavior (Cane et al., 2012). TDF is a synthesis of 33 theories of behavior and behavior change clustered into 14 (originally 12) domains (Cane et al., 2012). It has been applied across a range of healthcare systems and healthcare behaviors (Atkins et al., 2017). Two reviewers (EMD and MR) jointly coded the barriers and facilitators reported within the studies into domains of the TDF (the coding manual, including detailed descriptions of 14 domains and their underlying constructs, is included in the Supplementary Materials). Two codes were applied to the same extract if applicable. All codified extracts were then reviewed and discussed with a third reviewer with expertise in the TDF (JJF). In the next step of analysis the same reviewers (MR, EMD, JJF) used an inductive approach to identify subthemes (specific barriers and facilitators), within the coded domains of the TDF.

For the quantitative summary, the numbers of studies in which subthemes of barriers or facilitators were nominated or endorsed by participants were totalled. This do not reflect how many respondents cited the specific barriers/facilitator within studies. Subthemes that were reported in the majority (≥50%) of identified studies are referred to as ‘most commonly reported’ influences on ASP use.

## Results

### Search Results

**Figure 1:**
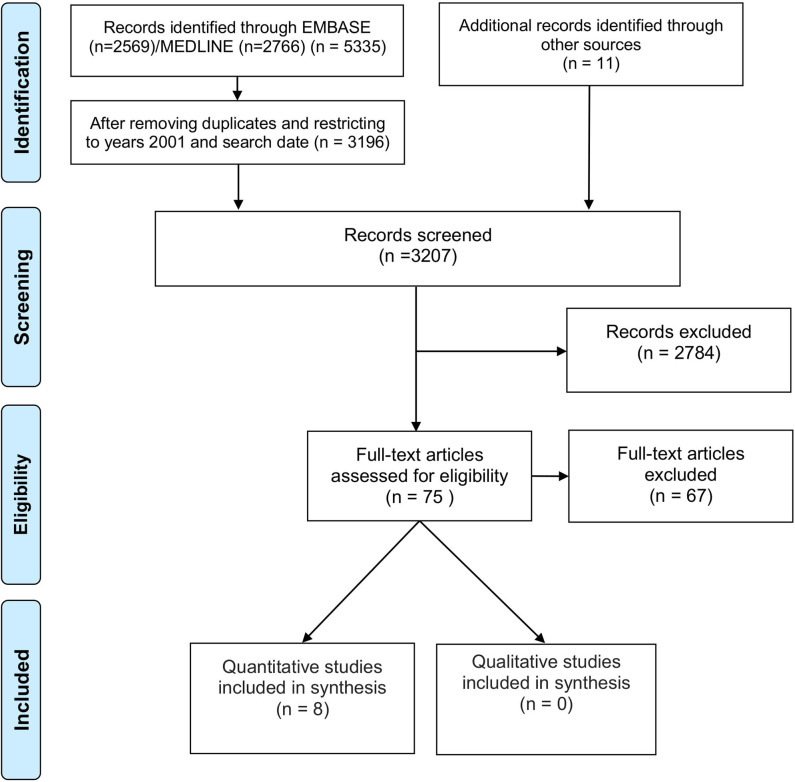
Study flow diagram.

The flow chart of the search and screening results is presented in Figure 1. Briefly, from 3,196 abstracts within the specified date limits, 75 full-text articles were screened for inclusion, of which 67 were excluded. Reasons for exclusion were: no full-text (*n* = 1), no original data (*n* = 11), not transnational (*n* = 15), study setting (*n* = 3), type of participants (*n* = 4), not an ASP (Charani et al., 2011), barriers or facilitators unreported (*n* = 18). Eight studies met the criteria and were included in this review (Itokazu et al., 2006; Johannsson et al., 2011; Howard et al., 2015; Bryant, 2015; Fleming et al., 2015; Livorsi et al., 2016; Wolf et al., 2016; Pulcini et al., 2017).

### Participants

All eight included studies were cross-sectional surveys. These studies included a total of 1849 participants, with sample sizes ranging from 14 (Bryant, 2015) to 660 (Howard et al., 2015). Two studies involving 704 participants reported individual participant level data (Itokazu et al., 2006; Johannsson et al., 2011). Five studies involving 1,057 institutions reported institutional level data (Howard et al., 2015; Bryant, 2015; Fleming et al., 2015; Livorsi et al., 2016; Wolf et al., 2016). One study reported country-level data and included national representatives of 36 countries (Pulcini et al., 2017). Two studies did not report numbers of respondents per country (Itokazu et al., 2006; Bryant, 2015) and one study provided incomplete information on a geographic location of participating institutions (Livorsi et al., 2016). The number of countries involved in each study ranged from 2 to 36, but overall participants from the North America, Europe and Australasia had the stronger representation in the identified studies. Participants were members or associates of established special interest groups or designated hospital representatives or ASP experts in charge at their hospitals. The characteristics of included studies and participants are presented in Table 1.

**Table 1:** Description of the included studies.

**References**	**Continent (country)**	**Response rate**	**Sample size**	**Design (origin)**	**Setting (number of institutions)**	**Participants (*n*)**	**Unit of analysis**	**Ongoing ASP**	**Reported ASP strategies (% actual)**
Itokazu et al., 2006	North America (USA and Canada)	88.5% (further 27.8% were excluded)	Total: 233 USA: NC Canada: NC	Electronic and postal survey (NC)	Teaching acute care hospital (NC)	Infectious disease pharmacists SIDP members (233)	Participants	99%	Education (88), prospective chart review (82), retrospective chart review (71), closed formulary (76), prior written or verbal approval (69), clinical practice guidelines (67), formal infectious diseases consultation (62), antibiotic switch (50), automatic stop order (47), antibiotic order form (30)
Johannsson et al., 2011	North America (USA and Canada)	50% (further 9.8% were excluded)	Total: 471 USA: 464 Canada: 7	Electronic survey (NC)	Community, teaching, city or county, veteran's affair hospitals caring for inpatients (NC)	Infectious disease physicians SHEA member (471)	Participants	61%	Formulation restriction pre-authorization and audit and feedback, education, guidelines and clinical pathways, conversion protocol, dose optimization, streamlining or automatic dose adjustment, time-sensitive stop orders, antimicrobial order forms, and antimicrobial cycling; (NC)
Bryant, 2015	Oceania (Australia; North and South Islands of New Zealand)	NC	Total: 14 Australia: NC North and South Islands of New Zealand: NC	Online survey (amended national survey, authors' ample ASP expertise)	Children's hospitals (*n* = 7), or hospitals with a large majority of adults (6) and one hospital with a majority of children plus a maternity unit (1)	Paediatric infectious disease physician (12), paediatrician (1), antimicrobial stewardship pharmacist (1)	Institution	64.3%	Treatment guidelines, education, selective susceptibility reporting, and point of-care interventions, approval for restricted antimicrobials, audit of antimicrobial use, monitoring of antimicrobial resistance; (NC)
Fleming et al., 2015	Europe (UK and Republic of Ireland)	Total: 36.4% Ireland: 73% UK: 32.7%	Total: 277 Ireland: 51 UK: 226	Postal survey (literature based, clinicians validated, piloted)	Ireland: private (15) and public (36) hospitals; UK: public hospitals (226)	Specialist antimicrobial pharmacists (NC), hospital pharmacists in charge (NC)	Institution	96.4%	Three most common strategies: empirical treatment of common infections, surgical prophylaxis and gentamicin protocol (NC)
Howard et al., 2015	Europe (26 countries), Oceania (2), Africa (10), Asia (14), North America (5), South and Central America (12)	? (9.8% were further excluded)	Total: 660 Europe: 361 Oceania: 30 Africa: 44 Asia: 25 N. America: 49 South and Central America: 44	Online survey (literature based, opinion leaders validated, piloted)	Tertiary teaching (319), district or general (161), community or private hospitals (56)	Hospital designated representatives (660)	Institution	58%	Treatment guidelines, surgical prophylaxis guidelines, closed formulary, reserve antibiotics needing authorization by indication, infectious diseases or microbiology advice by telephone or on ward rounds, dose optimization on request, intravenous-to-oral switch guidance, review of intravenous therapy at Day 3, systematic advice for bacteraemia by infectious diseases or microbiology, care bundles, automatic stop or review policy, pre-authorized pharmacy-driven dose optimization, separate antimicrobial chart or section, inflammatory markers to prevent initiation of antibiotics or to stop antibiotics early, restrictions on access by pharmaceutical representatives, antibiotic cycling; (NC)
Livorsi et al., 2016	USA and elsewhere	28.4%	Total: 61 USA: 52 Not reported a geographic location or institutional affiliation: 9	Online survey (NC)	Acute-care inpatient hospitals that participated in the SRN (61)	Physician (48), pharmacist (10), physician or pharmacist (3), SRN members engaged in prospective audit and feedback	Institution	NA	Prospective audit and feedback (100)
Wolf et al., 2016	North America (USA, Mexico, Canada); Oceania (Australia and NZ)	37.4% (4.9% were further excluded)	Total: 97 Australasia: 18 N. America: 72 Not reported a geographic location or institutional affiliation: 7	Online survey (literature search, a focus group)	Institutions that care for paediatric haematology, oncology and bone marrow transplant population (45)	ID physicians (55), fellows (13), clinical pharmacists (29), PIDS conference attendees or other relevant	Institution	91.1%	Clinical guideline development (80), dose optimization (78), resistance monitoring (76), prospective audit with feedback (71), monitoring of cultures (67), clinician education (64), encouraging oral switch (62), audit with delayed feedback (29), antibiotic cycling (9)
Pulcini et al., 2017	Europe (35 countries)^¥^ and Asia (Israel)	94.7%	Total: 36 Europe: 35 Israel	Online survey (literature search, ASP specialists validated)	Inpatient and outpatient care institutions- mainly tertiary university hospitals (NC)	EUCI (11) or EUCAST (13) members and appointed national representatives (12)	Country	NA	Selective reporting of antibiotic susceptibility test results (NC)

*EUCIC, European Committee on Infection Control; EUCAST, European Committee on Antimicrobial Susceptibility Testing; NC, not clear, SHEA, the Society of Healthcare Epidemiology of America; SRN, the Healthcare Epidemiology of America Research Network; PIDS, the Paediatric Infectious Diseases Society; SIDP, Society of Infectious Diseases Pharmacists*.

¥ *Austria, Azerbaijan, Belgium, Bosnia, Bulgaria, Croatia, Czech Republic, Denmark, Estonia, Finland, France, Germany, Greece, Hungary, Iceland, Ireland, Israel, Italy, Kosovo, Latvia, Macedonia, The Netherlands, Norway, Poland, Portugal, Romania, Russia, Serbia, Slovakia, Slovenia, Spain, Sweden, Switzerland, Turkey, UK, Ukraine*.

### Characteristics of Antibiotic Stewardship Programmes

Six studies explored barriers or facilitators to specific ASPs (Itokazu et al., 2006; Johannsson et al., 2011; Howard et al., 2015; Bryant, 2015; Wolf et al., 2016). Between 58% and 99% of respondents were from an institution with an ongoing ASP. Two studies referred to specific antibiotic stewardship strategies: audit and feedback (Livorsi et al., 2016), and selective reporting of antibiotic susceptibility test (AST) results (Pulcini et al., 2017). Respondents in the study on audit and feedback had to be engaged in this strategy to be eligible for participation (Livorsi et al., 2016). One study was restricted to ASP in pediatric oncology and bone marrow transplant (Wolf et al., 2016). Moreover, in one study (Bryant, 2015) pediatric hospitals accounted for half of included hospitals.

### Measures of Barriers and Facilitators

Seven studies examined barriers to antibiotic stewardship programmes or strategies (Itokazu et al., 2006; Johannsson et al., 2011; Howard et al., 2015; Bryant, 2015; Livorsi et al., 2016; Wolf et al., 2016; Pulcini et al., 2017) and one study reported possible facilitators (Fleming et al., 2015). One study asked participants to report solutions they employed to address experienced barriers, but findings related to this question were not reported (Pulcini et al., 2017). None of the studies explored the impact of health system factors (e.g., public vs. private healthcare systems). Three studies considered the impact of country context on reported barriers and facilitators (Howard et al., 2015; Fleming et al., 2015; Wolf et al., 2016). Five studies used closed-ended questions (i.e., a multiple-selection list of options) on barriers with prelisted response options (Itokazu et al., 2006; Johannsson et al., 2011; Howard et al., 2015; Bryant, 2015; Wolf et al., 2016). Three studies used open-ended questions to identify barriers and/or facilitators, one reported identified themes only (Livorsi et al., 2016) and two reported both themes and exemplary quotations (a qualitative component) (Howard et al., 2015; Fleming et al., 2015). Amongst studies that used closed-ended questions on barriers, two did not detail methods of questionnaire design (Itokazu et al., 2006; Johannsson et al., 2011), one used an amended questionnaire from a previously conducted survey based on literature search and expert opinion (Bryant, 2015), two searched literature, of which one also used expert advice (Howard et al., 2015) and one a focus group (Wolf et al., 2016). Only one of the three studies that used open-ended questions on barriers reported the method of data analysis (Pulcini et al., 2017), namely the framework method proposed by Flottorp et al. (2013). Overall, comprehensiveness and precision of the methods of how studies identified barriers and facilitators used was limited.

### Methodological Quality of Included Studies

Details of the Mixed Methods Appraisal Tool (MMAT) (Pluye et al., 2011) quantitative descriptive subsection scoring are presented for each study in Table S1. All included studies fulfilled the two screening criteria in the MMAT, suggesting that further methodological appraisal was feasible. All eight studies used an appropriate sampling strategy. The main methodological limitation was appropriateness (i.e., clear origin or known validity or standard measurement) of methods of assessing barriers and facilitators, with six studies not meeting this criterion (details in Table S1 footnote). Six studies scored negative or unclear on adequate response rate and four on representativeness, raising concerns of possible selection bias.

### Qualitative Synthesis

All barriers and facilitators were coded from the eight included studies into theoretical domains of the TDF. A summary of identified themes and subthemes of influences is presented in Table 1. Results of this coding can be found in Table S2. Data extracts coded into subthemes of TDF domains can be found in Table S3. Ten of the 14 domains of the TDF were present in the results reported in the eight studies (as presented in Table 2—“*Optimism*,” “*emotion*,” “*memory, attention and decision processes*” and “*beliefs about capabilities*” were not present in any of the results reported). Subthemes within each domain are described below, commencing with the domain with the largest number of subthemes.

**Table 2:** A summary of barriers (B) and facilitators (F) to implementing an antibiotic stewardship programme (ASP) or an ASP-supporting strategy.

**Theoretical domains framework–domains**	**Subthemes (Table S3) within each domain derived from coded data (Table S2)**	**No. of studies**
Environmental context and resources	(B) Lack of key personnel (e.g., infectious disease clinicians, pharmacy staff, microbiologist)	6
	(B) Problems with data and information systems (e.g., inadequate information technology, lack of dedicated IT assistant, lack of good quality data, and resources to utilize it)	6
	(B, F) The influence of adequacy of financial resources	4
	(B) Lack of time	3
	(B) Inadequate supply of laboratory provisions	1
	(B) Problem of limited antibiotic options available in settings with prevalent multi drug resistant bacteria	1
Goals	(B) Other higher priority initiatives hindering the ASP's use	4
Social influences	(B) Resistance from medical staff	3
	(B, F) The influence of clinical leadership (e.g., pharmacists, infectious diseases physicians, senior clinicians)	3
	(B) Lack of leadership from hospital administration	3
	(B) Poor communication, including interpersonal, within teams (e.g., inconsistency or conflict) and between private and public sectors	3
	(B) Perceived unhelpful attitudes of oncology clinicians	1
Behavioural regulation	(B, F) The influence of local guidelines and clinical practice protocols	2
	(F) Electronic prescribing as a mean to effectively change prescribing patterns by providing easier and quicker feedback	1
	(B) Lack of national and/or international standards required for a specific antibiotic stewardship strategy	1
	(B) Lack of standards for measuring performance of a specific antibiotic stewardship intervention	1
Knowledge	(B) Lack of knowledge of patient test or results	3
	(B) Lack of knowledge about ASPs (e.g., due to poor education or inevitable loss of knowledge due to high staff turnover)	2
	(B) Lack of knowledge of current use of antibiotics	1
Beliefs about consequences	(B) Lack of certainty about usefulness of an ASP or a specific antimicrobial stewardship strategy	2
	(B) ASP clinicians' belief in competing consequences of managing infections in different patient groups acting as a barrier	1
	(F) Focussing ASPs efforts on serious infectious disease as a mean to improving effectiveness of ASPs	1
Social/professional role and identity	(B) ASP derived jurisdiction gives antimicrobial stewardship clinicians limited power or authority	1
	(B) Uncertainties around overlapping responsibilities between multiple infectious diseases groups within a hospital	1
Intentions	(B) Lack of willingness to change	1
Reinforcement	(B) A specific antimicrobial stewardship strategy not being covered by a reimbursement system	1
Skills	(B) Medical professionals lacking relevant skills for a specific antimicrobial stewardship strategy (e.g., training in clinical microbiology)	1

#### Environmental Context and Resources

There was a perceived paucity of funding (Johannsson et al., 2011; Howard et al., 2015; Pulcini et al., 2017) and accordingly securing financial resources to develop and implement ASPs was mentioned as a facilitator (Fleming et al., 2015).

Insufficient pharmacist and clinician time allocated to ASP activities was also reported to hinder ASP efforts (Itokazu et al., 2006; Livorsi et al., 2016; Wolf et al., 2016). Conceptually related to this issue was a reported shortage of key personnel (Itokazu et al., 2006; Johannsson et al., 2011; Howard et al., 2015; Bryant, 2015; Livorsi et al., 2016; Pulcini et al., 2017), such as dedicated infectious disease clinicians, pharmacists or pharmacy staff, and microbiologists.

A range of barriers related to data and information systems availability and support were identified (Itokazu et al., 2006; Johannsson et al., 2011; Howard et al., 2015; Livorsi et al., 2016; Wolf et al., 2016) that resulted in poor access to patient information (Johannsson et al., 2011; Howard et al., 2015; Pulcini et al., 2017). Participants cited problems of inadequate quality of clinical data on the current use of antimicrobials (Livorsi et al., 2016) and insufficient data analysis resources (Wolf et al., 2016). Given the absence of dedicated information technology staff to support the selective reporting of AST results, additional technical support to manage the data was required. Lack of such support was seen to increase the workload for information technology staff (Pulcini et al., 2017).

Setting- and context-specific barriers included a lack of a reliable supply of laboratory provisions (i.e., shortage of laboratory materials) for selective reporting of antibiotic susceptibility testing (Pulcini et al., 2017), and the challenge of limited availability of antibiotic options faced by ASPs in hospital settings where multi-drug resistant bacteria are prevalent (Pulcini et al., 2017).

#### Social Influences

Interpersonal processes among healthcare professionals (including communication, cooperation and leadership) influenced ASP implementation. Poor communication was reported (Livorsi et al., 2016; Wolf et al., 2016; Pulcini et al., 2017), including within infectious disease and ASP teams (Wolf et al., 2016), between staff (e.g., antibiotic stewardship and pediatric oncology clinicians) (Wolf et al., 2016) and between public and private hospital systems (Pulcini et al., 2017). Studies also reported a lack of cooperation and even resistance from medical staff (Itokazu et al., 2006; Johannsson et al., 2011; Howard et al., 2015; Livorsi et al., 2016). An unsatisfactory relationship between antibiotic stewardship clinicians working in pediatric oncology settings and pediatric oncology clinicians was characterized by role conflict and lack of trust or shared beliefs (Wolf et al., 2016). Finally, a lack of leadership from hospital administrators (Itokazu et al., 2006; Howard et al., 2015; Bryant, 2015) and a lack of clinical leadership from pharmacists and infectious diseases clinicians were perceived to be barriers (Itokazu et al., 2006; Bryant, 2015). Participants suggested that the latter could be overcome by introducing a microbiologist team leader to facilitate the establishment of an antibiotic stewardship team (Fleming et al., 2015).

#### Behavioral Regulation

Availability of adequate guidance documents or recommendations at all levels (strategy-specific national and international, local, and setting-specific) influenced implementation of ASPs. A lack of national or international clinical practice guidelines to set professional standards for applying the selective reporting of AST results acted as a barrier (Pulcini et al., 2017). The selective reporting of AST results was reported to be particularly difficult in complicated cases (e.g., polymicrobial infections, severe infections, pharmacodynamics or pharmacokinetics factors) (Pulcini et al., 2017). As susceptibility testing occurs *in vitro*, this difficulty might be related to a need for specific guidance with accounting for many *in vivo* factors (e.g., pharmacokinetics factors) that can influence treatment success. Depending on the stage of ASP implementation, finalizing local guidelines on existing ASP efforts and developing implementation strategies was reported to be potentially helpful with implementing ASPs (Fleming et al., 2015). In pediatric oncology settings, insufficient input of antibiotic stewardship clinicians into clinical practice guidelines, and oncology clinicians following externally derived treatment pathways, were reported to be barriers to stewardship (Wolf et al., 2016).

Influences on behavioral regulation related to audit and feedback were also identified. A lack of performance metrics needed for audit and feedback was reported (Livorsi et al., 2016). Developing processes for the implementation of audit and feedback was cited to be potentially beneficial for implementation of ASPs (Fleming et al., 2015). To optimize feedback needed for adjusting prescribing patterns and to improve patient monitoring, the introduction of an electronic prescribing system was felt to be possibly useful (Fleming et al., 2015).

#### Knowledge

Studies indicated that limited access to relevant education (Bryant, 2015; Pulcini et al., 2017) and high level of turnover of junior staff was felt to be associated with an inevitable leakage and loss of ASP knowledge (Bryant, 2015). Insufficient knowledge about antibiotic resistance or clinical microbiology are examples of gaps in scientific background reported by participants (Pulcini et al., 2017). Lack of knowledge about patient clinical data (e.g., insufficiency of patient clinical data in a laboratory and delays in obtaining results) (Johannsson et al., 2011; Howard et al., 2015; Pulcini et al., 2017) and the current use of antimicrobials (Livorsi et al., 2016) were also identified.

#### Beliefs About Consequences

A lack of awareness among hospital administrators about the current value of ASPs was reported to hinder the delivery of functional and effective stewardship (Johannsson et al., 2011). Similarly, some unawareness of the value of the selective reporting of AST results and conflicting evidence on its usefulness, effectiveness or applicability were felt to impede its implementation (Pulcini et al., 2017).

Participants expressed the opinion that effectiveness of ASP efforts would further benefit from narrowing focus to serious infections, extended spectrum beta-lactamase-producing organisms, or carbapenem- and vancomycin-resistant organisms (Fleming et al., 2015).

#### Social/Professional Role and Identity

Uncertainty around division of responsibilities between multiple infectious diseases professional groups was identified, but only in one study, and its participants ranked it as the least common barrier (Johannsson et al., 2011).

#### Goals

The selective reporting of AST results was reported to be hindered by a lack of support in current ASP guidelines (Pulcini et al., 2017). Studies also reported other higher priority initiatives competing with establishing and maintaining ASPs (Johannsson et al., 2011; Howard et al., 2015; Pulcini et al., 2017). Populations other than immunocompromised hosts having higher priority in antibiotic stewardship programmes, was considered to be an important barrier by antibiotic stewardship clinicians (Wolf et al., 2016).

#### Intentions

A lack of willingness to change behavior was felt to hinder ASP implementation efforts (Bryant, 2015).

#### Reinforcement

A lack of incentives to use selective reporting of AST was reported to impede its implementation (Pulcini et al., 2017).

#### Skills

It was felt that local professionals are generally lacking relevant skills (especially physicians' clinical microbiology skills) needed for the selective reporting of AST results (Pulcini et al., 2017).

### Quantitative Summary

The most commonly reported (≥4 out of 8 studies) influences on ASP implementation were coded into the domain “*environmental context and resources”*: problems with data and information systems (e.g., inadequate information technology, lack of dedicated information technology assistance, lack of good quality data and resources to utilize it), lack of key personnel (e.g., infectious disease clinicians, pharmacy staff, microbiologist) and inadequacy of financial resources. In addition, other higher priority initiatives hindering the implementation of ASPs were coded into the domain “*goals*”) (Table 1).

### Country Context

One paper in this review (Wolf et al., 2016) found no effect of continent when comparing North American and Australasian institutions. Another included paper (Howard et al., 2015) concluded that a lack of funding or personnel and a lack of information technology or ability to acquire data (all coded into the domain “*environmental context and resources”*), followed by prescriber opposition (“*social influences”*) or other higher priorities (“*goals*”) were the top barriers to implementing an ASP, uniformly across all continents except for Africa, for which information technology was ranked as the main barrier. In hospitals that planned to develop an ASP, the main barrier was a lack of funding, except in South America, where a lack of awareness on the part of the hospital administration (“*social influences*”) that implies a lack of leadership from hospital administration, was the key barrier stated (Howard et al., 2015). “*Behavioural regulation”* strategies such as finalizing local guidelines on existing ASP efforts, developing processes for the implementation of ASP strategies and introducing an electronic prescribing system to adjust prescribing patterns were key strategic enablers of ASPs suggested by the UK, but not Irish respondents (Fleming et al., 2015). Ring-fencing of financial resources (“*environmental context and resources*”) and a need for microbiologist leadership (“*social influences*”) were mentioned by respondents from Ireland, but not from the UK (Fleming et al., 2015).

## Discussion

In response to calls for improved understanding of contextual influences on the implementation of ASP in hospitals (World Health Organization, 2015; Davey et al., 2017; Hulscher and Prins, 2017), we conducted a systematic review of transnational studies on reported barriers and facilitators to implementation of ASP in hospitals. Except for one study, most of the data comes only from developed countries (North America, Europe and Australasia). Reported barriers and facilitators were coded using the TDF, a framework based on behavioral theories. None of the included studies used behavioral theory explicitly to identify barriers and facilitators, reiterating the problem of behavioral approaches being underutilized in antibiotic stewardship studies (Charani et al., 2011; Rawson et al., 2017) and highlighting the importance of efforts to enable their widespread use (Atkins et al., 2017). The most commonly reported influences on ASP implementation included problems with data and information systems, lack of key personnel and financial resources. Another commonly reported barrier was the effect of conflicting priority initiatives. Between-country differences in the order of importance of specific influences on ASP implementation efforts warrant further investigation.

The main methodological weakness of included studies concerned the methods used in these studies to identify barriers and facilitators. Five studies used a list of options for barriers compiled by study authors and three studies used open-ended questions, hence barriers and facilitators were not comprehensively captured. It is unclear to what extent methods of assessment affected types of reported barriers. Two of three studies with high scores on quality of measurement of barriers were strategy- and context-specific and this possibly enabled participants to recall specific episodes or information more accurately. There were difficulties with quality of reporting, including the use of vague wording, incomplete descriptions and limited space dedicated to reporting barriers and facilitators. Frontline hospital workers involved in ASPs were rarely represented.

The review itself has its strength and limitations. By using TDF, a well-operationalized, multi-level implementation determinant framework derived from theory, we synthesized generic learning from diverse studies on dissimilar, context-specific multi-component ASPs, which may be useful to research teams designing future large-scale evaluation efforts. Other frameworks promote knowledge synthesis about what works, where and why, across multiple contexts, such as the Consolidated Framework For Implementation Research (CFIR) (Damschroder et al., 2009). However, given all included studies involved interventions to change antibiotic prescribing behavior, a behavioral approach using TDF was a suitable means of providing a high level of elaboration for contextual influences related to both individual-level change (provider behavior) and collective-level constructs. With a recognized need for the use of behavioral theory approaches (Davey et al., 2017; Rzewuska et al., 2019), the high level of transparency in our reporting of the review methods enhances existing guidance (Atkins et al., 2017). For example, a user less familiar with the TDF may find it easier to apply it, by following the outputs from each step of the analytic process reported in this paper. A limitation of the review is that, although we searched several databases using a comprehensive search strategy, we included only research published in English. Hence, the findings may not generalize beyond English language contexts.

The scope of our review has important implications. First, antibiotic stewardship involves different “actors,” including individuals (e.g., antibiotic prescribers), organizations and governments (Dyar et al., 2017). By focusing on hospital staff experiences with implementation of ASPs, we took a focused approach that is an organization level approach. Several factors are known to influence prescribing behavior in acute care hospital settings (Md Rezal et al., 2015), hence a need for understanding a context of antibiotic stewardship (Tamma et al., 2014). A methodological reason for taking a focused approach was that the framework method of analysis, thematic analysis and qualitative content analysis, involve categorization, which in turn requires data that is specified at a similar level (Gale et al., 2013). Second, we reasoned that there are qualities of the whole that cannot be reduced to the qualities of its parts and yet the nature of a part depends upon the whole in which it is embedded (Wagemans et al., 2012). As such we were interested in comparing multi-component ASPs (“whole”) with individual ASP-supporting interventions (“parts”). However, we aimed in this work to learn about implementation of ASPs, as opposed to conceptualizing individual behavior change interventions (“parts”). For an example of “why” and “how” the TDF can increase clarity and help to operationalise the individual intervention elements, we refer a reader to another paper published by the authors (Duncan et al., 2020). At last, to fully address the remaining uncertainties surrounding the value of antibiotic stewardship, we advocate for robust large-scale participatory collaborative evaluation research (Grimshaw et al., 2019; Rzewuska et al., 2019). Finding a balance between full and consistent implementation across multiple contexts, while providing the flexibility for individual sites to adapt the intervention as needed, is a major task (Damschroder et al., 2009). Multinational trials will face the design challenge of setting minimum conditions addressing differences between countries that are likely to generate unintended influences (barriers and facilitators) on outcomes and, thus, potentially hinder the generalisability and transferability of results. Therefore, we reasoned that commonalities captured through transnational studies would inform us about a broad scope of circumstances and characteristics that should be considered when facing early methodological issues, such as defining the scope of the evaluation and selecting study sites (Bryce et al., 2005). Country-level studies seem to be highlighting similar barriers. For example, quantitative analysis of data from three nationwide studies in the USA (Doron et al., 2013) and Australia (Chen et al., 2011; James et al., 2015) identified the same barriers as those reported in the current review. Chen and colleagues (Chen et al., 2011) concluded that barriers identified through quantitative and qualitative methods were alike. Overall, transnational studies were unlikely to use qualitative methods of data collection, which would seem to be more appropriate in the context of studies in which face-to-face methods of data collection are more feasible (Cotta et al., 2015; James et al., 2015).

Work presented in this review has the potential to inform local or regional initiatives to guide ASP implementation efforts. We have provided a detailed coding manual so that future initiatives may be informed by this behavioral approach.

There are several research implications of this review. There is an apparent need for a transnational mixed method study inclusive of low-, medium- and high-income countries, to identify barriers and facilitators to implementation of ASPs using a behavioral approach and to explore country, context and health systems differences. Assuming that optimizing ASP efforts may be effectively approached by addressing the commonly reported influences on ASP implementation, an appropriate next step is to identify strategies for optimizing ASPs that could, in turn, change prescribing behavior of frontline healthcare professionals. A wide range of behavior change approaches have been proposed, for example, education, persuasion, incentivisation, coercion, training, restriction, environmental restructuring, modeling or enablement (Michie et al., 2014).

## Conclusions

Despite a substantial research effort, and many quality improvement initiatives, there is still a poor evidence base to identify barriers and facilitators for establishing and maintaining ASPs transnationally, even in developed countries where most data comes from, and thus a poor basis for optimizing these large-scale quality improvement efforts to address what is a globally important problem. The reviewed here evidence suggests that these efforts will likely require taking into account the possibility of issues around inadequacy of information systems, unavailability of key personnel and funding, and the competition from other priority initiatives. To provide comprehensive generalizable evidence on barriers and facilitators to establishing and maintaining ASPs, a prospective transnational mixed methods study with hospital staff using behavioral theory may be worthwhile. For this purpose, we suggest using implementation frameworks, for example TDF is well-suited to design ASP interventions to enhance implementation and CFIR evaluation of the implementation of a specific ASP in multi-level contexts. This work enhances the evidence base to inform guidance by taking a behavioral approach to identify influences on ASP uptake.

## Data Availability Statement

Publicly available datasets were analyzed in this study. This data can be found here: EMBASE, MEDLINE.

## Author Contributions

MR, ED, and CR were involved in the conception and design of the review. MR and ED performed study selection, extracted data from the included studies, and critically appraised methodological quality of the included studies. MR, ED, and JF analyzed and interpreted the literature and drafted the manuscript. AM, KS, PD, JG, and CR revised the review critically for important intellectual content, contributed to data interpretation, and to the writing of the manuscript. All authors revised and approved the submission of the manuscript.

## Conflict of Interest

The authors declare that the research was conducted in the absence of any commercial or financial relationships that could be construed as a potential conflict of interest. The handling editor declared a past co-authorship with several of the authors PD, as well as EC and FL, members of the JPIAMR Working Group.
