# Supplementary material for: Barriers and Facilitators to Implementation of Antibiotic Stewardship Programmes in Hospitals in Developed Countries: Insights From Transnational Studies
Source: Front Sociol. 2020 Jul 8;5:41. doi: 10.3389/fsoc.2020.00041 (PMC8022532; doi:10.3389/fsoc.2020.00041)
Supplement: Supplementary file 1 [file Table_1.docx]

| **Table S1. Methodological quality of studies assed with the MMAT criteria** | | | | | | | | |
| --- | --- | --- | --- | --- | --- | --- | --- | --- |
|  | **Itokazu, 2006** | **Johannsson, 2011** | **Bryant, 2015** | **Fleming, 2015** | **Howard, 2015** | **Livorsi, 2016** | **Wolf, 2016** | **Pulcini, 2017** |
| **Screening questions:** |  |  |  |  |  |  |  |  |
| Are there clear quantitative research questions (or objectives)? | Y | Y | Y | Y | Y | Y | Y | Y |
| Do the collected data allow the research question (objective) to be addressed? | Y | Y | Y | Y | Y | Y | Y | Y |
| **Quantitative descriptive criteria:** |  |  |  |  |  |  |  |  |
| Is the sampling strategy relevant to address the quantitative research question? | Y | Y | Y | Y | Y | Y | Y | Y |
| Is the sample representative of the population under study? | N | N | Y | Y | Y | N | Y | N |
| Are measurements appropriate (clear origin or validity known or standard measurement)? | N | N | U^a^ | U^b^ | Y | N | N^c^ | Y |
| Is there an acceptable response rate (60% or above)? | Y | N | U | N | U | N | N | Y |

Each item is scored on a nominal scale (Yes/No/Unclear)

^a^ Bryant, 2015: Authors referred the Victorian AMS survey for description of the measurement used, but there were marked differences in phrasing of reported barriers and original a multiple-selection list of barriers

^b^ Fleming et al., 2015: Authors used questions from two previous employed surveys, none of which mentioned question(s) to evaluate barriers and facilitators within methods sections

^c^ Wolf et al., 2016: Presented data was difficult to interpret, given respondents were asked to make attributions about other people’s beliefs and motivations and authors used vague wording of the questionnaire items (e.g. “ASP [“Antimicrobial Stewardship Program] believes that other populations have higher priority”).
