# Supplementary material for: Barriers and Facilitators to Implementation of Antibiotic Stewardship Programmes in Hospitals in Developed Countries: Insights From Transnational Studies
Source: Front Sociol. 2020 Jul 8;5:41. doi: 10.3389/fsoc.2020.00041 (PMC8022532; doi:10.3389/fsoc.2020.00041)
Supplement: Supplementary file 2 [file Table_2.docx]

**Table S2. All analysed barriers (B) and facilitators (F) to implementing an antibiotic stewardship programme (ASP) or an ASP-supporting strategy**

| TDF domains | Exact quotes from the included studies [our comment] | Study | Applicability |
| --- | --- | --- | --- |
| Behavioural regulation | 1. “Locally we need to finalise guidelines and then begin to develop our audit and feedback processes. (UK)“ 2. “I think we have a comprehensive AMS which was identified by the SHA [Strategic Healthcare Authority] as a role model in the South East and I can see the main key strategy is to make sure all the trusts know how to implement DoH [Department of Health] Guidelines. (UK)” 3. “Electronic prescribing would make monitoring much easier and feedback immediate and effective in changing prescribing patterns. (UK)“ | Fleming, 2015^F^ | Generic |
|  | 1. “Insufficient ID or ASP input into local clinical practice guidelines” [Inclusion of antimicrobial stewardship guidance in internally derived clinical practice guidelines is a barrier]   (B) “Oncology clinicians follow externally derived collaborative group protocols” [Inclusion of antimicrobial stewardship guidance in externally derived collaborative group protocols is a barrier] | Wolf, 2016^G^ | Generic |
|  | 1. “Lack of national or international guidelines on AST results selective reporting (e.g. Each laboratory applies its own strategy for selective reporting of AST results, or does not use selective reporting at all)” 2. “Difficult applicability to complicated cases (e.g. Polymicrobial infections, PK/PD factors [pharmacodynamics/ pharmacokinetics], severe infections and other factors make selective reporting difficult to use in some patients)” | Pulcini, 2017^B^ | Strategy specific |
|  | 1. “Lack of stewardship [performance] metrics” [required for conducting audit and feedback] | Livorsi, 2016^E^ | Strategy specific |
| Beliefs about consequences | (B) “Administration not aware of value of ASP” | Johannsson, 2011^C^ | Generic |
|  | 1. “ASP does not have enough expertise in managing infections in immunocompromised hosts” [An ASP has a limited applicability to immunosuppressed hosts] | Wolf, 2016^G^ | Context specific |
|  | 1. “Lack of agreement (e.g. Some experts have doubts regarding usefulness or applicability of selective reporting)” 2. “Lack of awareness, familiarity and engagement (e.g. Professionals’ awareness of antibiotic resistance and usefulness of selective reporting is low)” | Pulcini, 2017^B^ | Strategy specific |
|  | 1. “Meeting the challenge posed by emergent multidrug resistant organisms e.g. carbapenem resistant enterococci, in the face of the paucity of new classes of antimicrobial agents. (Ireland)” 2. “Targeting and interventions to reduce carbapenemase producing organisms e.g. carbapenem review rounds to rationalise empiric use of carbapenems (UK)” 3. “Monitoring ESBL [Extended Spectrum Beta-Lactamases] and VRE [vancomycin-resistant enterococci] organisms. (UK)” | Fleming, 2015^F^ | Context specific |
| Competing goals | 1. “Other higher-priority clinical initiatives” | Johannsson, 2011^C^ | Generic |
|  | 1. “Other higher priority initiatives” | Howard, 2015^D^ | Generic |
|  | 1. “ASP believes that other populations [than paediatric oncology patients] have higher priority” [ASPs having higher priority populations than immunosuppressed hosts acting as a barrier] | Wolf, 2016^G^ | Context specific |
|  | 1. “Other more basic priorities (e.g. Quality control and standardisation of testing procedures are still the main issue)” | Pulcini, 2017^B^ | Strategy specific |
| Goal setting | 1. “Lack of priority (e.g. Selective reporting has not been included in the national/local AMS programmes)” | Pulcini, 2017^B^ | Strategy specific |
| Environmental context and | 1. “Complex implementation in areas with high MDR [multiple drug resistant] bacteria prevalence (e.g. A high prevalence of MDR pathogens limits the number of available antibiotic options)” | Pulcini, 2017^B^ | Context specific |
| Resources | (B) “Dedicated pharmacist time” (also coded at ‘*social influences*’)   1. “ID physician participation” (also coded at ‘*social influences*’) 2. “Pharmacy administration” (also coded at ‘*social influences*’) 3. “Inadequate computer linkage between the pharmacy and microbiology laboratory” | Itokazu, 2006^H^ | Generic |
|  | 1. “Lack of funding or personnel” 2. “Lack of information technology [to] support [an ASP] and/or inability to get [patient] data” (also coded at ‘*knowledge*’) | Johannsson, 2011^C^ | Generic |
|  | 1. “Lack of dedicated pharmacy staff” 2. “Lack of dedicated medical staff” | Bryant, 2015^A^ | Generic |
|  | 1. “Lack of resources, including limited time and personnel” 2. “The need for more rigorous informatics support” 3. “Lack of high-quality data on appropriate antimicrobial use” (also coded at ‘*knowledge*’) | Livorsi, 2016^E^ | Strategy specific |
|  | 1. “Insufficient data analysis resources” 2. “Insufficient clinician time assigned to antimicrobial stewardship” 3. “Lack of electronic prescribing” | Wolf, 2016^G^ | Generic |
|  | 1. “Lack of personnel or funding” 2. “Lack of information technology [to] support [an ASP] and/or inability to get [patient] data” (also coded at ‘*knowledge*’) | Howard, 2015^D^ | Generic |
|  | 1. “Lack of human resources (e.g. Human resources in the laboratory are insufficient to guarantee the availability of results 24 h/day and 7 days/week in case of clinician request)” 2. “Lack of dedicated IT (e.g. Available IT does not assist the microbiologist in selective reporting and makes it an additional workload)” 3. “Lack of regular supply of laboratory materials (e.g. Shortage of laboratory materials makes it difficult to organise AST according to guidelines)” 4. “Significant economic constraints on healthcare budget (e.g. There are significant financial problems that are prioritised in health system planning)” 5. “Lack of communication (e.g. Patient clinical data available in the laboratory are insufficient or Informing clinicians on hidden results is difficult and time consuming)” (also coded at ‘*knowledge*’) | Pulcini, 2017^B^ | Strategy specific |
|  | 1. “It will be difficult to progress programs without ring fencing of resources needed to implement and develop antibiotic programmes. (Ireland)” | Fleming, 2015F | Generic |
| Intentions | 1. “Lack of willingness to change” | Bryant, 2015^A^ | Generic |
| Knowledge | 1. “Lack of education”   (B) [High level of] “transient junior staff” [that implies an inevitable leakage and loss of knowledge] | Bryant, 2015^A^ | Generic |
|  | 1. “Lack of awareness, familiarity and engagement (e.g. Professionals’ awareness of antibiotic resistance and usefulness of selective reporting is low)” (also coded at ‘beliefs about consequences’) 2. “Lack of capability (e.g. Scientific background and capabilities of local professionals are insufficient)” (also coded at ‘*skills*’)   (B) “Lack of physicians trained in clinical microbiology (e.g. Microbiologists are mainly technicians with biology background, clinical microbiology [training] is not available as medical specialty)” (also coded at ‘*skills*’)  (B) “Lack of communication (e.g. Patient clinical data available in the laboratory are insufficient or informing clinicians on hidden results is difficult and time consuming)” (also coded at ‘*environmental context and resources’*) | Pulcini, 2017^B^ | Strategy specific |
|  | 1. “Lack of information technology [to] support [an ASP] and/or inability to get [patient] data” (also coded at ‘*environmental context and resources’*) | Johannsson, 2011^C^  Howard, 2015^D^ | Generic |
|  | 1. “Lack of high-quality data on appropriate antimicrobial use” [A lack of knowledge of the current use of antimicrobials] | Livorsi, 2016^E^ | Strategy specific |
| Reinforcement | 1. “Lack of recognition by the reimbursement system (e.g. Reimbursement system does not recognise or support selective reporting)” | Pulcini, 2017^B^ | Strategy specific |
| Skills | 1. “Lack of capability (e.g. Scientific background and capabilities of local professionals are insufficient)” (also coded at ‘*knowledge*’) 2. “Lack of physicians trained in clinical microbiology (e.g. Microbiologists are mainly technicians with biology background, clinical microbiology [training] is not available as medical specialty)”(also coded at ‘*knowledge*’) | Pulcini, 2017^B^ | Strategy specific |
| Social influences | 1. “Medical staff cooperation” 2. “ID physician participation” (also coded at ‘*environmental context and resources*’) 3. “Support of the hospital administrator” 4. “Dedicated pharmacist time” (also coded at ‘*environmental context and resources*’) | Itokazu, 2006^H^ | Generic |
|  | 1. “Opposition from prescribers” 2. “Other specialties antagonized by ASP” | Johannsson, 2011^C^ | Generic |
|  | 1. “Lack of leadership by executive and senior clinicians” 2. “Lack of enforcement [by hospital management]” | Bryant, 2015^A^ | Generic |
|  | 1. “Opposition from prescribers” 2. “Administration not aware of ASP” [therefore unable to enforce an ASP] | Howard, 2015^D^ | Generic |
|  | 1. “Physician [negative] attitudes about antimicrobials” 2. “Prescribers resistant to feedback” 3. [Poor] “Communication” | Livorsi, 2016^E^ | Strategy specific |
|  | 1. “Not enough communication with oncology clinicians” 2. “Inconsistency or conflict within the infectious diseases or ASP team”   (B) “Oncology clinicians are concerned about loss of autonomy”   1. “Oncology clinicians are more motivated by fear of rare adverse outcomes than long-term risks of antimicrobial use” 2. “Oncology clinicians don't show confidence in ASP/ID clinicians”   (B) “Oncology clinicians do not believe that their antimicrobial use leads to antimicrobial resistance”  (B) “Oncology clinicians are confident in their antibiotic knowledge or current antimicrobial strategies” [Reliance of paediatric oncology clinicians on their knowledge and current strategies to manage infections]  (B) “Oncology clinicians are not motivated by reducing financial costs (e.g. Use of expensive antibiotics)” | Wolf, 2016^G^ | Context specific |
|  | 1. “Lack of collaboration from the private system (e.g. Private laboratories prefer full reporting because it requires less qualified personnel and implies less workload)” | Pulcini, 2017^B^ | Strategy specific |
|  | 1. “Despite repeated attempts to put an Antimicrobial Stewardship team in place it has not happened. We need a Microbiologist to push things forward. (Ireland)” [A need for introducing a microbiologist team leader to facilitate the establishment of an antimicrobial stewardship team] | Fleming, 2015^F^ | Context specific |
| Social/professional role and identity | 1. [Lack of clarity about division of responsibilities between]“Multiple infectious disease groups within facility” | Johannsson, 2011^C^ | Generic |
|  | 1. “ASP does not have enough power or authority” [ASP derived jurisdiction gives antimicrobial stewardship clinicians limited power or authority] | Wolf, 2016^G^ | Context specific  Generic |

AMS, antimicrobial stewardship; ASP, antimicrobial stewardship program; AST, antibiotics susceptibility test; ID, infectious diseases;

**Methods of assessing barriers and facilitators**: ^A^ a multiple-selection list of reasons for slow implementation of an ASP; ^B^ an open-ended question on barriers faced when implementing a selective reporting of antibiotic test results; ^C^ a multiple-selection list of barriers (with an option none) to current, planned, hypothetical functional and effective ASPs, ranked from 1 least to 7 most common; ^D^ a multiple-selection list of barriers (with options none and other) to current and planned functional and effective ASPs, selected if applicable; ^E^ an open-ended question on the biggest barriers your ASP faces; ^F^ an open ended question on key strategic issues around an ASP; ^G^ two questions with multiple-selection list of barriers to implementation of an ASP and oncology clinician, scored using 5-point scale (not at all important to very important) and one question on inclusion of antimicrobial stewardship guideline oncology treatment protocols a berries with two yes and one no response options available; ^H^ a multiple-selection list of aspects of programmatic support for an ASP, rated as adequate or inadequate.
