# Supplementary material for: Barriers and Facilitators to Implementation of Antibiotic Stewardship Programmes in Hospitals in Developed Countries: Insights From Transnational Studies
Source: Front Sociol. 2020 Jul 8;5:41. doi: 10.3389/fsoc.2020.00041 (PMC8022532; doi:10.3389/fsoc.2020.00041)
Supplement: Supplementary file 3 [file Table_3.docx]

| **Table S3. Subthemes of barriers and facilitators derived from coded data (Table S2).** | |  |
| --- | --- | --- |
| **TDF domain: subthemes** | **Exact quotes from the included studies coded at the specific domain and then subtheme [comments]** | **Barrier or Facilitator** |
| **Behavioural regulation:** |  |  |
| The influence of local guidelines and clinical practice protocols | “Insufficient ID or ASP input into local clinical practice guidelines” (Wolf et al., 2016) [Inclusion of antimicrobial stewardship guidance in internally derived clinical practice guidelines is a barrier] | Barrier |
|  | “Oncology clinicians follow externally derived collaborative group protocols” (Wolf et al., 2016) [Inclusion of antimicrobial stewardship guidance in externally derived collaborative group protocols is a barrier] | Barrier |
|  | “Locally we need to finalise guidelines and then begin to develop our audit and feedback processes. (UK)“ (Fleming et al., 2015) | Facilitator |
|  | “I think we have a comprehensive AMS which was identified by the SHA [Strategic Healthcare Authority] as a role model in the South East and I can see the main key strategy is to make sure all the trusts know how to implement DoH [Department of Health] Guidelines. (UK)”(Fleming et al., 2015) | Facilitator |
| Electronic prescribing as a mean to effectively change prescribing patterns by providing easier and quicker feedback | “Electronic prescribing would make monitoring much easier and feedback immediate and effective in changing prescribing patterns. (UK)“ (Fleming et al., 2015) | Facilitator |
| Lack of national and/or international standards required for a specific antibiotic stewardship strategy | “Lack of national or international guidelines on AST results selective reporting (e.g. Each laboratory applies its own strategy for selective reporting of AST results, or does not use selective reporting at all)”(Pulcini et al., 2017) | Barrier |
|  | “Difficult applicability to complicated cases (e.g. Polymicrobial infections, PK/PD [pharmacodynamics/ pharmacokinetics] factors, severe infections and other factors make selective reporting difficult to use in some patients)”(Pulcini et al., 2017) | Barrier |
| Lack of standards for measuring performance of a specific antibiotic stewardship strategy | “Lack of stewardship [performance] metrics” [required for conducting audit and feedback] (Livorsi et al., 2016) | Barrier |
| **Beliefs about consequences:** |  |  |
| ASP clinicians’ belief in competing consequences of managing infections in different patient groups acting as a barrier | “ASP does not have enough expertise in managing infections in immunocompromised hosts” (Wolf et al., 2016) [An ASP has a limited applicability to immunosuppressed hosts] | Barrier |
| Lack of certainty about usefulness of an ASP or a specific antimicrobial stewardship strategy | “Administration not aware of value of ASP” (Johannsson et al., 2011) | Barrier |
|  | “Lack of agreement (e.g. Some experts have doubts regarding usefulness or applicability of selective reporting)” (Pulcini et al., 2017) (also coded at ‘knowledge’) | Barrier |
|  | “Lack of awareness, familiarity and engagement (e.g. Professionals’ awareness of antibiotic resistance and usefulness of selective reporting is low)” (Pulcini et al. 2017) (also coded at ‘knowledge’) | Barrier |
| Focussing ASPs efforts on serious infectious disease as a mean to improving effectiveness of ASPs | “Meeting the challenge posed by emergent multidrug resistant organisms e.g. carbapenem resistant enterococci, in the face of the paucity of new classes of antimicrobial agents. (Ireland)”(Fleming et al., 2015) | Facilitator |
|  | “Targeting and interventions to reduce carbapenemase producing organisms e.g. carbapenem review rounds to rationalise empiric use of carbapenems (UK)” (Fleming et al., 2015) | Facilitator |
|  | “Monitoring ESBL [Extended Spectrum Beta-Lactamases] and VRE [vancomycin-resistant enterococci] organisms. (UK)” (Fleming et al., 2015) | Facilitator |
| **Environmental context and resources:** |  |  |
| Problem of limited antibiotic options available in settings with prevalent multi drug resistant bacteria | “Complex implementation in areas with high MDR [multiple drug resistant] bacteria prevalence (e.g. A high prevalence of MDR pathogens limits the number of available antibiotic options)” (Pulcini et al., 2017) | Barrier |
| The influence of adequacy of financial resources | “Lack of funding or personnel”(Johannsson et al., 2011, Howard et al., 2015) | Barrier |
|  | “Significant economic constraints on healthcare budget (e.g. There are significant financial problems that are prioritised in health system planning)”(Pulcini et al., 2017) | Barrier |
|  | “It will be difficult to progress programs without ring fencing of resources needed to implement and develop antibiotic programmes. (Ireland)” (Fleming et al., 2015) | Facilitator |
| Lack of time | “Dedicated pharmacist time” (Itokazu et al. 2006) (also coded at ‘*social influences*’) | Barrier |
|  | “Lack of resources, including limited time and personnel” (Livorsi et al., 2016) | Barrier |
|  | “Insufficient clinician time assigned to antimicrobial stewardship” (Wolf et al., 2016) | Barrier |
| Lack of key personnel (e.g. infectious disease clinicians, pharmacy staff, microbiologist) | “ID physician participation” (Itokazu et al., 2006) (also coded at ‘*social influences*’) | Barrier |
|  | “Pharmacy administration” (Itokazu et al., 2006) (also coded at ‘*social influences’*) | Barrier |
|  | “Lack of funding or personnel” (Johannsson et al., 2011, Howard et al., 2015) | Barrier |
|  | “Lack of dedicated pharmacy staff” (Bryant, 2015) | Barrier |
|  | “Lack of dedicated medical staff”(Bryant, 2015) | Barrier |
|  | “Lack of resources, including limited time and personnel” (Livorsi et al., 2016) | Barrier |
|  | “Lack of human resources (e.g. Human resources in the laboratory are insufficient to guarantee the availability of results 24 h/day and 7 days/week in case of clinician request)” (Pulcini et al., 2017) | Barrier |
| Problems with data and information systems (e.g. inadequate information technology, lack of dedicated IT assistant, lack of good quality data and resources to utilize it) | “Inadequate computer linkage between the pharmacy and microbiology laboratory” (Itokazu et al., 2006) | Barrier |
|  | “Lack of information technology [to] support [an ASP] and/or inability to get [patient] data” (Johannsson et al., 2011, Howard et al., 2015) (also coded at ‘*knowledge*’) | Barrier |
|  | “The need for more rigorous informatics support” (Livorsi et al., 2016) | Barrier |
|  | “Lack of high-quality data on appropriate antimicrobial use” (Livorsi et al., 2016) (also coded at ‘*knowledge*’) | Barrier |
|  | “Insufficient data analysis resources” (Wolf et al., 2016) | Barrier |
|  | “Lack of electronic prescribing” (Wolf et al., 2016) | Barrier |
|  | “Lack of dedicated IT (e.g. Available IT does not assist the microbiologist in selective reporting and makes it an additional workload)” (Pulcini et al., 2017) | Barrier |
|  | “Lack of communication (e.g. Patient clinical data available in the laboratory are insufficient or Informing clinicians on hidden results is difficult and time consuming)”(Pulcini et al., 2017) (also coded at ‘*knowledge*’) | Barrier |
| Inadequate supply of laboratory provisions | “Lack of regular supply of laboratory materials (e.g. Shortage of laboratory materials makes it difficult to organise AST according to guidelines)” (Pulcini et al., 2017) | Barrier |
| **Goals:** |  |  |
| Other higher priority initiatives hindering the ASP’s use | “Other higher-priority clinical initiatives”(Johannsson et al., 2011) | Barrier |
|  | “Other higher priority initiatives” (Howard et al., 2015) | Barrier |
|  | “ASP believes that other populations [than paediatric oncology patients] have higher priority” (Wolf et al., 2016) [ASPs having higher priority populations than immunosuppressed hosts acting as a barrier] | Barrier |
|  | “Other more basic priorities (e.g. Quality control and standardisation of testing procedures are still the main issue)” (Pulcini et al., 2017) | Barrier |
|  | “Lack of priority (e.g. Selective reporting has not been included in the national/local AMS programmes)” (Pulcini et al., 2017) | Barrier |
| **Intentions:** |  |  |
| Lack of willingness to change | “Lack of willingness to change” (Bryant, 2015) | Barrier |
| **Knowledge:** |  |  |
| Lack of knowledge about ASPs (e.g. due to poor education or inevitable loss of knowledge due to high staff turnover) | “Lack of education” (Bryant, 2015) | Barrier |
|  | [High level of] “transient junior staff” [that implies an inevitable leakage and loss of knowledge] (Bryant, 2015) | Barrier |
|  | “Lack of awareness, familiarity and engagement (e.g. Professionals’ awareness of antibiotic resistance and usefulness of selective reporting is low)” (also coded at ‘*beliefs about consequences*’) (Pulcini et al., 2017) | Barrier |
|  | “Lack of capability (e.g. Scientific background and capabilities of local professionals are insufficient)” (Pulcini et al., 2017) (also coded at ‘*skills*’) | Barrier |
|  | “Lack of physicians trained in clinical microbiology (e.g. Microbiologists are mainly technicians with biology background, clinical microbiology [training] is not available as medical specialty)” (Pulcini et al., 2017) (also coded at ‘*skills*’) | Barrier |
| Lack of knowledge of patient test or results | “Lack of communication (e.g. Patient clinical data available in the laboratory are insufficient or informing clinicians on hidden results is difficult and time consuming)” (Pulcini et al., 2017)(also coded at ‘*environmental context and resources*’) | Barrier |
|  | “Lack of information technology [to] support [an ASP] and/or inability to get [patient] data” (Howard et al., 2015, Johannsson et al., 2011) (also coded at ‘*environmental context and resources*’) | Barrier |
| Lack of knowledge of current use of antibiotics | “Lack of high-quality data on appropriate antimicrobial use” (Livorsi et al., 2016) (also coded at ‘*environmental context and resources’*) | Barrier |
| **Reinforcement:** |  |  |
| A specific antimicrobial stewardship strategy not being covered by a reimbursement system | “Lack of recognition by the reimbursement system (e.g. Reimbursement system does not recognise or support selective reporting)”(Pulcini et al., 2017) | Barrier |
| **Skills:** |  |  |
| Medical professionals lacking relevant skills for a specific antimicrobial stewardship strategy (e.g. training in clinical microbiology) | “Lack of capability (e.g. Scientific background and capabilities of local professionals are insufficient)” (Pulcini et al., 2017) (also coded at ‘*knowledge*’) | Barrier |
|  | “Lack of physicians trained in clinical microbiology (e.g. Microbiologists are mainly technicians with biology background, clinical microbiology [training] is not available as medical specialty)” (Pulcini et al., 2017) (also coded at ‘*knowledge*’) | Barrier |
| **Social influences:** |  |  |
| Resistance from medical staff | “Medical staff cooperation”(Itokazu et al., 2006) | Barrier |
|  | “Opposition from prescribers” (Johannsson et al., 2011, Howard et al., 2015) | Barrier |
|  | “Other specialties antagonized by ASP” (Johannsson et al., 2011) | Barrier |
|  | “Physician’s [negative] attitudes about antimicrobials” (Livorsi et al., 2016) | Barrier |
|  | “Prescribers resistant to feedback” (Livorsi et al., 2016) | Barrier |
| The influence of clinical leadership (e.g. pharmacists, ID physicians, senior clinicians) | “ID physician participation” (also coded at ‘*environmental context and resources*’) (Itokazu et al., 2006) | Barrier |
|  | “Dedicated pharmacist time” (also coded at ‘*environmental context and resources*’) (Itokazu et al., 2006) | Barrier |
|  | “Lack of leadership by executive and senior clinicians” (Bryant, 2015) | Barrier |
|  | “Despite repeated attempts to put an Antimicrobial Stewardship team in place it has not happened. We need a Microbiologist to push things forward. (Ireland)” (Fleming et al., 2015) [A need for introducing a microbiologist team leader to facilitate the establishment of an antimicrobial stewardship team] | Barrier |
| Lack of leadership from hospital administration | “Support of the hospital administrator” (Itokazu et al., 2006) | Barrier |
|  | “Lack of enforcement [by hospital management]” (Bryant, 2015) | Barrier |
|  | “Administration not aware of ASP” [therefore unable to enforce an ASP] (Howard et al., 2015) | Barrier |
| Poor communication, including interpersonal, within teams (e.g. inconsistency or conflict) and between private and public sectors | [Poor] “communication” (Livorsi et al. 2016) | Barrier |
|  | “Not enough communication with oncology clinicians” (Wolf et al., 2016) | Barrier |
|  | “Inconsistency or conflict within the infectious diseases or ASP team” (Wolf et al., 2016) | Barrier |
|  | “Lack of collaboration from the private system (e.g. Private laboratories prefer full reporting because it requires less qualified personnel and implies less workload)”(Pulcini et al., 2017) | Barrier |
| Perceived unhelpful attitudes of oncology clinicians | “Oncology clinicians are concerned about loss of autonomy” (Wolf et al., 2016) | Barrier |
|  | “Oncology clinicians are more motivated by fear of rare adverse outcomes than long-term risks of antimicrobial use” (Wolf et al., 2016) | Barrier |
|  | “Oncology clinicians do not believe that their antimicrobial use leads to antimicrobial resistance” (Wolf et al., 2016) | Barrier |
|  | “Oncology clinicians don't show confidence in ASP/ID clinicians” (Wolf et al., 2016) | Barrier |
|  | “Oncology clinicians are not motivated by reducing financial costs (e.g. Use of expensive antibiotics)” (Wolf et al., 2016) | Barrier |
|  | “Oncology clinicians are confident in their antibiotic knowledge or current antimicrobial strategies” (Wolf et al., 2016) [Reliance of paediatric oncology clinicians on their knowledge and current strategies to manage infections] | Barrier |
| **Social/professional role and identity:** | |  |
| Uncertainties around overlapping responsibilities between multiple infectious diseases groups within a hospital | [Lack of clarity about division of responsibilities between] “Multiple infectious disease groups within facility” (Johannsson et al., 2011) | Barrier |
| ASP derived jurisdiction gives antimicrobial stewardship clinicians limited power or authority | “ASP does not have enough power or authority” (Wolf et al., 2016) | Barrier |
| AMS, antimicrobial stewardship; ASP, antimicrobial stewardship program; AST, antibiotics susceptibility test; ID, infectious diseases. | | |
