# Supplementary material for: Barriers and Facilitators to Implementation of Antibiotic Stewardship Programmes in Hospitals in Developed Countries: Insights From Transnational Studies
Source: Front Sociol. 2020 Jul 8;5:41. doi: 10.3389/fsoc.2020.00041 (PMC8022532; doi:10.3389/fsoc.2020.00041)
Supplement: Supplementary file 4 [file Table_4.docx]

Coding instructions:

- The purpose of this work is to categorize barriers and facilitators to setting or implementing antibiotic stewardship programs (ASPs) in inpatient hospital settings using the Theoretical Domains Framework (Cane, O’Connor & Michie 2012).
- Note that definitions of the domains and the underlying constructs are from Michie et al. (Michie, S, Atkins, L, West, R 2014), Cane et al. (Cane, O’Connor & Michie 2012) and the final coding manual used to identify barriers and facilitator to delivery of an individualized dialysis temperature in the MyTEMP trial (Presseau et al. 2017) unless otherwise indicated (i.e., “MR”). The “Decision Rule” column provided to aid the coding into domain has been inspired by Presseau et al.’s manual (Presseau et al. 2017).
- Code all responses both positive and negative, actual and hypothetical and include ‘no’ answers when relevant.
- Can code highlighted text to multiple domains if needed.
- Code all extracted relevant text into each domain (i.e. keep the context information to show how the extract is associated with a domain), but also highlight the specific sections of the extract specifically related to the domain.
- Wherever needed (e.g. a coded extract is not self-explanatory) use the definitions provided in this codebook to justify coding.

Special attention needed when differentiating between:

- - ‘Beliefs about Capabilities’ versus ‘Professional/Social Role and Values’
  - ‘Professional/Social Role and Values’ versus ‘Social influences’
  - ‘Behavioural Regulation’ versus ‘Environmental Context and Resources - Resources’ and ’Reinforcement’
  - ‘Beliefs about Consequences’ versus ‘Optimism’ and ‘Emotion’

Cane, J., O’Connor, D. & Michie, S. 2012, "Validation of the theoretical domains framework for use in behaviour change and implementation research", *Implementation science,* vol. 7, no. 1, pp. 37.

Michie, S, Atkins, L, West, R 2014, *The Behaviour Change Wheel: A Guide to Designing Interventions,* 1st edn, Silverback Publishing, London.

Presseau, J., Mutsaers, B., Al-Jaishi, A.A., Squires, J., McIntyre, C.W., Garg, A.X., Sood, M.M. & Grimshaw, J.M. 2017, "Barriers and facilitators to healthcare professional behaviour change in clinical trials using the Theoretical Domains Framework: a case study of a trial of individualized temperature-reduced haemodialysis", *Trials,* vol. 18, no. 1, pp. 227.

| **Domain** | **Theoretical constructs represented within each domain** | **Decision Rule** | **Exemplary exact quotes from the included studies** |
| --- | --- | --- | --- |
| **Domains of the TDF that were present in the results reported in the eight studies:** | | | |
| **Behavioural Regulation** Anything aimed at managing or changing objectively measured actions  *Do they have a system that could be used for monitoring whether or not they have carried out [an ASP]?*  *Do they have a set of rules to regulate how activities related to an ASP are being carried out in their hospitals? (MR)* | Self-monitoring: *A method used in behavioural management in which individuals keep a record of their behaviour, especially in connection with efforts to changes or regulate the self; a personality trait reflecting an ability to modify one’s behaviour in response to a situation.*  Breaking habit: *To discontinue a behaviour or sequence of behaviours that is automatically activated by relevant situational cues.*  Action planning: *The action or process of forming a plan regarding a thing to be done or a deed.* | Consider coding to this domain:   - Descriptions or statements of the influence or importance of system regulatory rules (e.g. local protocols) to standardize the delivery of ASP.   *Note:* Can be actual or hypothetical. Include ‘no’ answers.   - Descriptions of self-regulatory strategies already in place that would influence the implementation of an ASP (e.g. a description of current steps or activities undertake to optimize antibiotic prescribing). - Descriptions of coping plans, systems, problem solving scripts or strategies used (that could be used) in response to overcome resistance or streamline the use an ASP on a systemic or individual level. Focus on strategies to ease the engagement in the behaviour (not incentives).   *Key words***:** protocols, manuals, monitoring | *“****Oncology clinicians follow externally derived collaborative group protocols****”*    *“****Electronic prescribing would make monitoring much easier and feedback immediate and effective in changing prescribing patterns.*** *“*  *“Lack of national or international guidelines on AST results selective reporting (e.g.* ***Each laboratory applies its own strategy for selective reporting of AST results, or does not use selective reporting at all****)”* |
| **Beliefs about Consequences**  Acceptance of the truth, reality or validity about outcomes of a behaviour in a given situation  *What do they think will happen if they will do it?* | Beliefs: *The thing believed; the proposition or set of propositions held true.*  Outcome expectancies: *Cognitive, emotional, behavioural, and affective outcomes that are assumed to be associated with future or intended behaviour. These assumed outcomes can either promote or inhibit future behaviours.*  Characteristics of outcome expectancies:  *Characteristics of the cognitive, emotional and behavioural outcomes that individuals believe are associated with future or intended behaviours and that are believed to either promote or inhibit these behaviours. These include whether they are sanctions/rewards, proximal/distal, valued/not valued, probable/ improbable. Salient/not salient, perceived risks or threats.*  Anticipated regret: *A sense of the potential negative consequences of a decision that influences the choice made: for example an individual may decide not to make an investment because of the feelings associated with an imagined loss.*  Consequents: *An outcome behaviour in a given situation.* | Consider coding to this domain:  Awareness of the value or usefulness of an ASP for healthcare provision.  Specific benefits and unintended consequences (outcomes) of delivering an ASP for patient outcomes, health care professionals and unit.  *Note:* Consequences may include toxicity, the selection of pathogenic organisms and the emergence of resistance.  Descriptions of concerns regarding efficiency of ASPs.  Beliefs about treatment outcomes – both theoretical and based on experience due to using an ASP.  Potential long term risks and benefits of using an ASP (e.g. the impact on antimicrobial resistance).  Competing consequences of an ASP for different groups of patients, types of antimicrobials, settings.  Descriptions or statements of the importance of targeting specific groups of patients, types of antimicrobials, settings with ASP efforts - both theoretical and based on experience due to using an ASP. An underlying beliefs is that of theoretical or experienced greater effectiveness of APS effort in those subgroups.  Relative importance of risks and benefits associated with an ASP use (e.g. adverse outcomes in immunosuppressed patients versus long-term collateral damage).  Health care professionals talking about consequences of using an ASP for other health care professionals (e.g. an increased workload).  Frequently double-coded:  Descriptions of affects associated with beliefs about consequences (e.g. disliking an ASP strategy, feeling apprehensive about) (code also to ‘*Emotions*’)  Inappropriate coding to this domain:  Participants’ descriptions of how positive or negative they are regarding effectiveness of an ASP or an effective ASP being eventually delivered (code to ‘*Optimism*’ instead) | “Lack of awareness, familiarity and engagement (e.g. **Professionals’ awareness of** antibiotic resistance and **usefulness of selective reporting is low**)”  “**Targeting and interventions to reduce carbapenemase producing organisms** e.g. carbapenem review rounds to rationalise empiric use of carbapenems)” |
| **Environmental**  **Context and Resources**  Any circumstance  of a person’s situation or environment that discourages or encourages the development of skills and abilities, independence, social competence, and adaptive behaviour  *What are the things in their environment that influence what they do and how do they influence?* | Environmental stressors*: External factors in the environment that cause stress.*  Resources or material resources: *Commodities and human resources used in enacting a behaviour.*  Organizational culture or climate: *A distinctive pattern of thought and behaviour shared by members of the same organization and reflected in their language, values, attitudes, beliefs and customs.*  Salient events or critical incidents: *Occurrences* *that one judges to be distinctive, prominent or otherwise significant.*  Person x environment interaction: *Interplay between the individual and their surroundings.*  Barriers and facilitators: *In psychological contexts, barriers or facilitators are mental, emotional or behavioural limitations or strengths in individuals or groups.* | Consider coding to this domain   - Descriptions or statements of the influence of adequacy of available personnel time dedicated to delivering an ASP, funding, system support, expert personnel (e.g. microbiologist) and equipment (e.g. software, laboratory materials). - Descriptions of how environmental or contextual factors can affect the antibiotics use.   *Note*: Can be actual or hypothetical. Include ‘no’ answers.  *Key words:* resources, technical support, equipment, availability  Frequently double-coded:   - Adequacy of available information technology systems is likely to have implications for availability of information (code also to ‘*knowledge*’).   Availability of a key personnel (infectious disease clinician or pharmacist in charge) is likely to have implications for availability of required leadership (code also to ‘*social influences*’). | *“****Inadequate computer linkage between the pharmacy and microbiology laboratory****”*  *“****Lack of resources, including limited time and personnel****”*  *“****It will be difficult to progress programs without ring fencing of resources needed to implement and develop antibiotic programmes****.”*  *“****The need for more rigorous informatics support****”*  *“Complex implementation in areas with high MDR [multiple drug resistant] bacteria prevalence (e.g.* ***A high prevalence of MDR pathogens limits the number of available antibiotic options****)”* |
| **Goals**  Mental representations of outcomes or end states that an individual wants to achieve  *How important is what they do and does that influence whether or not they do it?*  *What standards are they trying to reach, how does that influence whether or not they do it?* | Goal priority: *Order of importance or urgency of end state toward which one is striving.*  *Goal/target setting: A process that establishes specific time-based behavioural targets that are measurable, achievable and realistic.*  Goals (autonomous or controlled): *The end state toward which one is striving: the purpose of an activity or endeavour. It can be identified by observing that a person ceases or changes their behaviour upon attaining this state; proficiency in a task to be achieved within a set period.*  Action planning: *The action or process of forming a plan regarding a thing to be done or a deed.*  Implementation intention: *The plan that one creates in advance of when, where and how one will enact a behaviour.* | Consider coding to this domain:  Competing goals: Descriptions of how setting or improving an ASP is (or is not) in conflict witch the current guidelines or local policies or is (or is not) in conflict with other objectives of the care provided or unit standards.  Goal setting: Descriptions of whether or not implementing an ASP is a priority (e.g. is (or is not) a part of guidelines or local policies currently used). | “Other more basic priorities (e.g. **Quality control and standardisation of testing procedures are still the main issue**)”  “**ASP [clinician] believes that other populations have higher priority**” |
| **Intention**  A conscious decision to perform a behaviour or a resolve to act in a certain way  *How does how inclined they are to do something influence whether they will do it?* | Stability of intentions: *Ability of one’s resolve to remain in spite of disturbing influences.*  Stages of Change model: *A model that proposes that behaviour change is accomplished through five specific stages.*  Trans-theoretical model and stages of change: *A five-stage theory to explain changes in people’s health behaviour. It suggests that change takes time, that different interventions are effective at different stages, and that there are multiple outcomes occurring across the stages.* | Consider coding to this domain:  Participant’s descriptions of how motivated they are to deliver an ASP.  Participant’s inclinations to change behaviour in order to apply an ASP.  Participant’s inclinations to apply an ASP to all patients.  Participants’ descriptions of when they are and are not inclined to apply an APS. Also code to other domains as necessary (i.e. ‘Beliefs about Consequences’)  *Note:* Indicator of intention must be explicit and not inferred. Focus on statements that directly reflect their intention and motivation and not to code the reasons for the intention.  Inappropriate coding to this domain:  This is different from how effective they think using an ASP will be or how efficient it is in managing infections (code to ‘*Beliefs About Consequences*’ instead). It’s also different from how much of a priority implementation of an ASP is for them (code to ‘*Goals*’ instead). | “**Lack of willingness to change**” |
| **Knowledge**  An awareness of the existence of something  *What do they know and how does that influence what they do?* | Knowledge (Including knowledge of condition or scientific rationale): *An awareness of the existence of something*  Procedural knowledge: *Knowing how to do something*  Knowledge of task environment: *Knowledge of the social and material context* | Consider coding to this domain:  Descriptions or statements of the influence of:   - Awareness of what ASPs are. - Awareness of core elements of an ASP and how it could/should be done. - Adequacy of scientific background in ASPs (e.g. antimicrobial resistance and effectiveness of ASP strategies). - Adequacy of access to information on patient microbiological test results. - Availability of data required to deliver effective ASPs (e.g. antibiotic usage).   Description or statements of what resources (e.g. information or data) is needed to deliver ASPs.  *Note:* Can be actual or hypothetical. Include ‘no’ answers.  *Key words:* awareness, scientific background, education, information, data access, knowing  Inappropriate coding to this domain:   - Descriptions or statements of the influence of hospital administration’s awareness of the existence of ASPs (code at ‘*Social influence’* instead).   Descriptions of how a resource (e.g. an electronic medical record system) could be used to regulate a behaviour (code at *Behavioural regulation’* instead). | *“Lack of communication (e.g.* ***Patient clinical data available in the laboratory are insufficient*** *or informing clinicians on hidden results is difficult and time consuming”*  *“Lack of awareness, familiarity and engagement (e.g.* ***Professionals’ awareness of antibiotic resistance and usefulness of selective reporting is low****)”* |
| **Reinforcement**  Increasing the probability of a response by arranging a dependent relationship, or contingency, between the response and a given stimulus  *How have their experiences (good and bad) of doing it in the past influence whether or not they do it?*  *Is there something that does or could encourage or discourage them to do it? (MR)* | Incentives: *An external stimulus, such as condition or object, that enhances or serves as a motive for behaviour.*  Punishment: *The process in which the*  *relationship between as response and some stimulus or circumstance results in the response becoming less probable; a painful, unwanted or undesired event or circumstance imposed as a penalty on a wrongdoer.*  Consequents: *An outcome of behaviour in a given situation.*  Reinforcement: *A process in which the frequency of a response is increased by a dependent relationship or contingency with a stimulus.*  Contingencies: *A conditional probabilistic relation between two events. Contingencies may be arranged via dependencies or they may emerge by accident.*  Sanctions: *A punishment or other coercive measure, usually administered by a recognized authority that is used to penalise and deter inappropriate or unauthorized actions.* | Consider coding to this domain:  Descriptions of the influence of positive and negative reinforcement for delivering an ASP.  Consider both the explicit and intrinsic reward.  The impact of the rationale for using an ASP on person’s motivation to deliver it.  *Note*: Can be actual or hypothetical. Include ‘no’ answers. | “Lack of recognition by the reimbursement system (e.g. **Reimbursement system does not recognise or support selective reporting**)” |
| **Skills**  An ability or proficiency acquired through practice  *Do they know how to do it?* | Skills: *An ability or proficiency acquired* *through training and/or practice.*  Skills development: *The gradual acquisition or advancement through progressive stages of an ability or proficiency acquired through training and practice.*  Competence: *One’s repertoire of skills, and ability especially as it is applied to a task or set of tasks.*  Ability: *Competence or capacity to perform a physical or mental act. Ability may be either unlearned or acquired by education and practice.*  Interpersonal skills: *An aptitude enabling a person to carry on effective relationships with others, such as an ability to cooperate, to assume appropriate social responsibilities or to exhibit adequate flexibility.*  Practice: *Repetition of an act, behaviour, or series of activities, often to improve performance or acquire a skill.*  Skills assessment: *A judgement of the quality, worth, importance. Level or value of an ability or proficiency acquired through training and practice.* | Consider coding to this domain:  Descriptions or statements of the influence or importance of:   - Psychological or physical ability to conduct a specific task related to an ASP acquired through training or practice.   *Note:* Can be actual or hypothetical. Include ‘no’ answers.  *Key words:* practice, training, experience, capability. | *“Lack of capability (e.g.* ***Scientific background*** *and capabilities* ***of local professionals are insufficient****)”* |
| **Social Influences**  Those interpersonal processes that can cause individuals to  change their thoughts, feelings, or behaviours  *What do others think of what they do?*  *Who are they and how does that influence what they do?* | Social pressure: the exertion of influence on a  person or group by another person or group  Social norms: *Socially determined consensual standards that indicate a) what behaviours are considered typical in a given context and b) what behaviours are considered proper in the context.*  Group conformity: *The act of consciously maintaining a certain degree of similarity to those in your general social circles.*  Social comparisons: *The process by which people evaluate their attitudes, abilities or performance relative to others.*  Group norms: *Any behaviour, belief, attitude or emotional reaction held to be correct or acceptable by a given group in society.*  *Social support: The apperception or provision of assistance or comfort to others, typically in order to help them cope with a variety of biological, psychological and social stressors. Support may arise from any interpersonal relationship in an individual’s social network, involving friends, neighbours, religious institutions, colleagues, caregivers of support groups.*  Power: *The capacity to influence others, even when they try to resist this influence.*  Intergroup conflict: *Disagreement or confrontation between two or more groups and their members. This may involve physical violence, interpersonal discord, or psychological tension.*  Alienation: *Estrangement from one's social*  *group; a deep seated sense of dissatisfaction with one's personal experiences that can be a source of lack of trust in one's social or physical environment or in oneself; the experience of separation between thoughts and feelings.*  Group identity: *The set of behavioural or personal characteristics by which an individual is recognizable [and portrays] as a member of a group.*  Modelling: *In developmental psychology the process in which one or more individuals or other entities serve as examples (models) that a child will copy.* | Consider coding to this domain:  Descriptions or statements of the influence of:   - Hospital management and other healthcare professionals on whether or not an ASP can be implemented. Can include positive modelling or unhelpful attitude (e.g. resistance to prescribe/set up an ASP). - Descriptions of patients’ emotions regarding antibiotic use. - The effectiveness of communication about ASP related behaviour, between members of clinical teams. - Unit-wide pattern of thought related to an ASP (considered social norms). - Perceptions of attitudes of other health care professionals.   *Note*: Can be actual or hypothetical. Include ‘no’ answers.   - Descriptions of who would be needed to someone else’s behaviour (e.g. leading or modelling through a positive example by a hospital administrator, ID clinicians, senior clinical staff). | *“Despite repeated attempts to put an Antimicrobial Stewardship team in place it has not happened.* ***We need a Microbiologist to push things forward****.”*  *“****Prescribers resistant to feedback****”*  *“****Support of the hospital administrator****”*  *“****Lack of leadership by executive and senior clinicians****”*  *“****Not enough communication with oncology clinicians****”* |
| **Social/Professional Role and Identity**  A coherent set of behaviours and displayed personal qualities of an individual in a social or work setting  *How does who they are as healthcare professionals influence whether they do something or not?*  *Is delivering an ASP compatible or in conflict with professional standard or identity?* | Professional identity: *The characteristics by which an individual is recognised relating to, connected with or befitting a particular profession.*  Professional role: *The behaviour considered appropriate for a particular kind of work or social position.*  Social identity: *The set of behavioural or personal characteristics by which an individual is recognizable [and portrays] as a member of a social group.*  Identity: *An individual’s sense of self defined by a) a set of physical and psychological characteristics that is not wholly shared with any other person and b) a range of social and interpersonal affiliations (e.g. ethnicity) and social roles.*  Professional boundaries: *The bounds or limits relating to, or connected with a particular profession or calling.*  Professional confidence: *An individual’s belief in his or her repertoire of skills and ability especially as it is applied to a task or set of tasks.*  Group identity: *The set of behavioural or personal characteristics by which an individual is recognizable [and portrays] as a member of a group.*  Leadership: *The processes involved in leading others, including organising, directing, coordinating and motivating their efforts toward achievement of certain group or organization goals.*  Organizational commitment: *An employee’s dedication to an organisation and wish to remain part of it. Organisational commitment is often described as having both an emotional or moral element and a more prudent element.* | Consider coding to this domain:   - Descriptions of who does what in a clinical team or an ASP team. Specifically what each different health care provider does (including what others do) as it relates to (or would relate to) the process of delivery of an ASP. - Descriptions of the influence of division of roles between groups with overlapping responsibilities in the delivery of ASP. - Description of how ASPs are in conflict (or are not) with standards or identity of a specific type of healthcare professional. - Implications of a character of a professional role for an opportunity to deliver an ASP (e.g. authority of an ASP clinician).   Inappropriate coding to this domain:   - Descriptions or statements of a need for someone else’s behaviour instead of details of a specific role (code to ‘*Social Influences’ instead*). - Descriptions or statements of the influence or impact of self-assurance in one’s capabilities and judgement rather than self-belief in an acquired set of skills (as a professional) and ability to apply those (code to ‘*Beliefs about Capability’ instead*). | “**Multiple infectious disease groups within facility**”  “**ASP [clinician] does not have enough power or authority**” |
| **Domains of the TDF that were NOT present in the results reported in the eight studies:** | | | |
| **Beliefs about Capabilities**  Acceptance of the truth, reality, or validity about an ability, talent or facility that a person can put to constructive use  *Do they think they can do what they should so and how does that influence whether they do it or not?* | Perceived competence: *An individual’s belief in her or her ability to learn and execute skills.*  Self-efficacy: *An individual’s capacity to act effectively to bring about desired results, as perceived by the individual.*  Perceived behavioural control: *An individual’s perception of the ease or difficulty of performing the behaviour of interest.*  Beliefs: *The thing believed; the proposition or set of propositions held true.*  Self-esteem: *The degree to which the qualities and characteristics contained in one’s self concept are perceived to be positive.*  Empowerment: *The promotion of the skills, knowledge and confidence necessary to take great control of one’s life as in certain educational or social schemes; the delegation of increase decision-making powers to individuals or groups in a society or organisation.*  Professional confidence: *An individual’s beliefs in his or her repertoire of skills, and ability, especially as it is applied to a task or set of tasks.* | Consider coding to this domain:  Descriptions of how easy or difficult it will or would be deliver an ASP.  Descriptions of how confident a physician feels to manage infections with or without an ASP. |  |
| **Emotion**  A complex reaction pattern, involving experiential, behavioural and physiological elements, by which the individual attempts to deal with a personally significant matter or event  *How do they feel about what they do and do those feelings influence what they do?* | Fear: *An intense emotion aroused by the detection of imminent threat, involving an immediate alarm reaction that mobilizes the organism by triggering a set of physiological changes.*  *Anxiety: A mood state characterized by apprehension and somatic symptoms of tension in which an individual anticipates impending danger, catastrophe or misfortune.*  *Affect: An experience or feeling of emotion, ranging from suffering to elation, from the simplest to the most complex sensations of feelings, and from the most normal to the most pathological emotional reactions.*  *Stress: A state of physiological or psychological response to internal or external stressors.*  *Depression: A mental state that presents with depressed mood, loss of interest or pleasure, feelings of guilt or low self-worth, disturbed sleep or appetite, low energy, and poor concentration.*  *Positive/negative affect: The internal feeling/state that occurs when a goal has/has not been attained. A source of threat has/has not been avoided, or the individual is/is not satisfied with the present state of affairs.*  *Burn-out: Physical, emotional or mental exhaustion, especially in one’s job or career, accompanied by decreased motivation, lowered performance and negative attitudes towards oneself and others.* | Consider coding to this domain:  Descriptions of emotions experienced by healthcare professionals or hospital administrators when setting/delivering an ASP. Can be positive or negative.  Descriptions of when healthcare professionals would be worried about the use of an ASP. Include ‘no’ answers.  Inappropriate coding to this domain:  Descriptions of patients’ emotions regarding  antibiotic use (code at ‘*Social Influences’* instead)  This is different from descriptions of concerns regarding efficiency of ASPs (code at *‘Beliefs about Consequences*’ instead) |  |
| **Memory, Attention and Decision Process**  The ability to retain information, focus selectively on aspects of the environment and choose between two or more alternatives  *Are tasks/processes related to [their ASPs] something that they usually do?* | Memory: *The ability to retain information or a representation of a past experience, based on the mental processes of learning or encoding retention across some interval of time, and retrieval or reactivation of the memory; specific information of a specific task.*  Attention: *A state of awareness in which the senses are focussed selectively on aspects of the environment and the central nervous system is in a state of readiness to respond to stimuli.*  Attention control: *The extent to which a person can concentrate on relevant cues and ignore all irrelevant cues in a given situation.*  Decision making: *The cognitive process of choosing between two or more alternatives, ranging from the relatively clear-cut to the complex.*  Cognitive overload/tiredness: *The situation in which the demands placed on a person by mental work are greater than a person’s mental abilities.* | Consider coding to this domain:   - Descriptions of when and/or why would it be easy to forget to conduct ASP related tasks, as well as reasons why they don’t think they would forget it.   Inappropriate coding to this domain:   - Descriptions of motives for not engaging in the ASP related behaviour due to underlying beliefs about validity of outcomes of the behaviour (code to ‘*Beliefs about Consequences’* instead). - Descriptions of the influence of confidence in their ability to engage in the target behaviour or alternative behaviour (code to ‘*Beliefs about capabilities*’ instead). |  |
| **Optimism**  The confidence that things will happen for the best or that desired goals will be attained  *How confident they are that the problem of implementation of [an ASP] will be solved?*  *How confident they are that responsible antibiotic use could be optimized using an ASP? (MR)*  *How confident they are that infections can be managed effectively in hospitals? (MR)* | Optimism: *The attitude that outcomes will be positive and that people’s wishes or aims will be ultimately fulfilled.*  Pessimism: *The attitude that things will go wrong and that people’s wishes or aims are unlikely to be fulfilled.*  Unrealistic optimism: *The inert tendency for humans to over-rate their own abilities and chances of positive outcomes compared to those of other people.* | Consider coding to this domain:  Participants’ descriptions of their level of optimism regarding the effectiveness of an ASP or an effective ASP being eventually delivered. Can be positive or negative answers and actual or hypothetical. |  |
